# The protective effects of Phoenixin-20 in tumor necrosis factor α (TNF-α)-induced cell senescence of rheumatoid arthritis fibroblast-like synoviocytes (FLS)

**DOI:** 10.18632/aging.205024

**Published:** 2023-11-28

**Authors:** Jinhua Yan, Ling Yao, Ying Tan, Yue Wang

**Affiliations:** 1 Department of Hematology and Rheumatology, The First Hospital of Nanchang, Nanchang, Jiangxi 330008, China; 2 Department of Gastroenterology, The First Hospital of Nanchang, Nanchang, Jiangxi 330008, China; 3 Department of Gerontology, The First Hospital of Nanchang, Nanchang, Jiangxi 330008, China; 4 Department of Rheumatology and Immunology, The First Hospital of Nanchang, Nanchang, Jiangxi 330008, China

**Keywords:** Phoenixin-20, rheumatoid arthritis, rheumatoid arthritis fibroblast-like synoviocytes, cell senescence, STAT6

## Abstract

Rheumatoid arthritis (RA) is an age-related joint destruction disease that markedly impacts the normal life of patients. Currently, the clinical treatment strategies are far from satisfactory with severe side effects. Cellular senescence of fibroblast-like synoviocytes (FLS) has been reported to be involved in the pathological process of arthritis, which may provide an important research direction for RA treatment. Phoenixin-20 (PNX-20) is a peptide targeting G-protein-coupled receptor 173 (GPR173) with promising anti-inflammatory properties. Our study will probe into the function of PNX-20 on tumor necrosis factor α (TNF-α)- induced rheumatoid arthritis (RA) FLS cell senescence to provide a theoretical basis for treating RA with PNX-20. RA-FLSs were handled with 10 ng/mL TNF-α, followed by introducing Phoenixin-20 (10, 20 nM) or not for 7 days. Enhanced release of inflammatory cytokines, increased proportion of senescence-associated β-galactosidase (SA-β-gal) positive cells, and declined telomerase activity were all observed in TNF-α-treated RA-FLSs, accompanied by a noticeable decline in the p21 and p53 level, which were notably reversed by 10 and 20 nM PNX-20. Furthermore, the increased signal transducer and activator of transcription 6 (STAT6) level observed in TNF-α-treated RA-FLSs were signally repressed by PNX-20. Moreover, the impact of PNX-20 on TNF-α-induced cellular senescence in RA-FLSs was abrogated by the overexpression of STAT6. Collectively, PNX-20 protected the TNF-α-induced cell senescence in RA-FLSs by downregulating STAT6. Based on these findings, we speculate that PNX-20 might be a promising agent for the treatment of RA.

## INTRODUCTION

Rheumatoid arthritis (RA), an autoimmune disease, is a common cause of joint destruction leading to disability, affecting approximately 1% of the adult population worldwide [1]. RA is characterized by slowness, inflammation, systemic progression, joint inflammation, joint space stenosis, cartilage degradation, and bone injury, synovial malformation and hyperplasia, and invasive pannus formation are reported to be the main pathological features of RA [2]. Morning stiffness, joint swelling and pain, joint abnormalities, low-grade fever, emaciation, and fatigue are the main clinical manifestations of RA [3]. In China, 5 million patients are reported to suffer from RA, with an incidence rate of 0.36% and a high disability rate [4]. Although non-steroidal anti-inflammatory drugs (NSAID), traditional antirheumatic drugs, novel biological agents, and glucocorticoids have been studied and used in the treatment of RA [5], there are still severe side effects, such as immune deficiency, fluid disorder, liver damage, and gastrointestinal disorder, that are claimed [6, 7]. Moreover, the overall efficacy of these drugs is far from satisfactory [8]. The pathological mechanism and pathogenic factors of RA remain unclear. Thus, there is an urgent need to explore the underlying mechanisms and explore safer alternative agents to improve inflammatory pathologic progression in RA patients. Fibroblast-like synoviocytes (FLS), as important cells in the synovial lining layer of joint tissue, are found activated in RA synovial cells and exhibit multiple tumorlike biological behaviors, such as invasion, migration, and excessive proliferation [9], which are not observed in other fibroblasts. RA FLS are key effector cells and play a key role in almost all aspects of RA progression [10]. Synovial hyperplasia is a typical feature of RA and is mainly caused by aggressive proliferation and apoptotic resistance of RA FLS, which contributes to the development of synovial inflammation and joint destruction [11]. Inhibition of proliferation and induction of apoptosis of RA FLS may be a feasible ideal strategy for RA treatment [12]. Recent studies have shown that the cell senescence of FLS is involved in the pathological process of arthritis [13, 14]. Furthermore, the senescence induced by chronic inflammation is found to participate in the progression of RA by impairing the immunomodulatory properties of synovial fluid mesenchymal stem cells [15]. Suppressing the cell senescence of RA FLS may be an important research direction for RA treatment.

Phoenixin (PNX) was first isolated from rat hypothalamus and bovine heart and has been identified in numerous mammals and non-mammals, which is expressed in various central and peripheral tissues and induces the activation of G-protein-coupled receptor (GPR173) [16]. PNX was speculated to play a role in multiple levels of the hypothalamus-pituitary-gonad (HPG) axis [17]. The amino acid sequence of PNX-20 is AGIVQEDVQPPGLKVWSDPF-NH2, which extends 6 amino acid peptides from the N-terminal co-expressed with PNX-14 [18, 19]. Recent studies have found that PNX-20 exerts a promising inhibitory effect on LPS-induced inflammatory response [20, 21].

However, it is unknown whether PNX-20 possesses a function in the pathophysiological characteristics of RA. Our study will explore the effect of PNX-20 on TNF-α-induced RA FLS cell senescence to provide a theoretical basis for treating RA with PNX-20.

## MATERIALS AND METHODS

### Cells and treatments

Human RA-FLSs were obtained from ATCC (HTX1834, USA) and were cultured in Dulbecco’s modified Eagle’s medium (DMEM) (Sigma-Aldrich, USA) supplemented with 10% fetal bovine serum (FBS) (Gibco, USA) and 1% Penicillin-Streptomycin, which were incubated at 37°C and 5% CO_2_. To obtain STAT6-overexpressed RA-FLSs, cells were transfected with a lentiviral STAT6 vector for 48 h, followed by evaluating the efficacy using Western blotting.

### Real-time polymerase chain reaction (RT-PCR) assay

Following different treatments, RA-FLSs were collected and lysed by Trizol reagent (Thermo Fisher Scientific, USA) to extract total RNAs. The isolated RNAs were quantified with NanoDrop One and transformed into cDNA using a commercial kit (MedChemExpress, USA), followed by performing the PCR reaction using the 2 × RealStar Fast SYBR qPCR Mix kit (GenStar, China) The gene expression was determined using the 2^−ΔΔCt^ method. The following primers were used in this study: GPR173: F: 5′-CAGCTAGTGGGAGGAAGCTGCT-3′; R: 5′-TGCTGAGCTACACCTGCAAATGGG-3′; SIRT6: F: 5′-GACAAGCTGGCCGAGCTGTACGGAAACAT-3′, R: 5′-ACAGCTCGGCCAGCTTGTCCCTGGGGA-3′; p53, F: 5′-GAAGACCCAGGTCCAGATGA-3′, R: 5′-CTCCGTCATGTGCTGTGACT-3′; P21: F: 5′-GCGCCATGTCAGAACCGGCTGG-3′, R: 5′-TTAGGGCTTCCTCTTGGAGA-3′; GAPDH: F, 5′-ACTGGCGTCTTCACCACCAT-3′, R, 5′-AAGGCCATGCCAGTGAGCTT-3′.

### Cell counting kit-8 (CCK-8) assay

RA-FLSs were inoculated in a 96-well plate and cultured in a 37°C incubator for 1 day. Then 10 μL CCK-8 solution (Sigma-Aldrich, USA) was added to each well at a final concentration of 10 μM, and incubated in a 37°C incubator for 2 h. The absorbance at 450 nm was determined with a microplate reader (LIUYI, China), and the cell viability was calculated.

### Enzyme-linked immunosorbent assay (ELISA)

The commercial kit for IL-6, IL-1β, and IL-8 (eBioScience, USA) was utilized for the detection, with instructions strictly followed. The prepared supernatants were added to a 96-well plate, incubated at 37°C for 30 min, and washed 5 times. After adding enzyme-labeled reagents, samples were incubated at 37°C for 30 min, followed by introducing chromogenic solutions A and B to be incubated at 37°C for 10 min. Following adding the termination solution, the optical density (OD) value was read to calculate the concentration of inflammatory factors.

### Senescence-associated β-galactosidase (SA-β-gal) staining assay

RA-FLSs were inoculated in a 24-well plate for 24 h and the cell culture medium was removed, followed by being washed once with PBS. Then, 1 mL β-galactosidase staining fixing solution was added, and fixed for 15 min. Cells were then introduced with 1 mL of staining working solution, followed by incubation at 37°C overnight. Images were taken under the optical microscope for observation and counting (Zeiss, German).

### The telomerase activity

RA-FLSs were lysed using the CHAPS buffer, followed by centrifugation at 15000 g for 1.5 h. Proteins were quantified with the bicinchoninic acid (BCA) (Sigma-Aldrich, USA) method. The activity of telomerase was then determined utilizing the TeloTAGGG Telomerase PCR ELISA PLUS kit (Roche, Switzerland), with instructions strictly followed. The TRAP-PCR reaction system, samples, and primers were introduced together, followed by conducting the quantification utilizing the RT-PCR assay.

### Western blotting assay

RA-FLSs were collected into a plate to extract total proteins, followed by performing the quantification with the BCA method. After separating utilizing the sodium dodecyl sulfate-polyacrylamide gel electrophoresis (SDS-PAGE) (12%), target proteins were moved to the poly-vinylidene fluoride (PVDF) membrane, followed by blocking with 5% non-fat milk. The primary antibodies against p53 (#AB_2860544, 1:2000, Sino Biological, China), p21 (1:1000, #ab109520, Abcam, USA), STAT6 (#ab32108, 1:1500, Abcam, USA), and β-actin (1:10000, #ab6276, Abcam, USA) were imported. Then, the membrane was rinsed and introduced with the secondary antibody (1:2000, #7074, 7076, Cell Signaling Technology, USA). The exposure of bands was conducted with the enhanced chemiluminescence (ECL) (Thermo Fisher Scientific, USA) solution. Western blot results were quantified using the software Image J. Firstly, the western blot bands were scanned and the backgrounds were subtracted. The target bands were then selected and the integrated intensity of each band was then calculated.

### Statistical analysis

Achieved data were presented as mean ± standard deviation (S.D.) and were analyzed with the GraphPad software. One-way analysis of variance (ANOVA) method with Tukey’s test was applied for comparison. *P* < 0.05 was regarded as a significant difference.

## RESULTS

### GPR173 was downregulated in TNF-α-treated RA-FLSs

**Figure 1:**
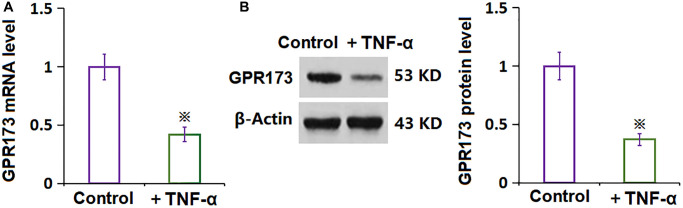
**The expression of GPR173 was decreased in TNF-α-treated rheumatoid arthritis fibroblast-like synoviocytes (RA-FLSs).** (**A**) mRNA of GPR173 as measured by real-time PCR; (**B**) Protein of GPR173 as measured with western blots analysis (^※^*P* < 0.01 vs. vehicle group, *N* = 6).

To explore the potential function of PNX-20 on TNF-α-stimulated RA-FLSs, the level of GPR173, the target protein of PNX-20, was checked. The GPR173 level was found markedly repressed in RA-FLSs after the stimulation of TNF-α (Figure 1A, 1B), implying a function deficit of GPR173 in TNF-α-treated RA-FLSs.

### The determination of PNX-20 concentration in RA-FLSs

**Figure 2:**
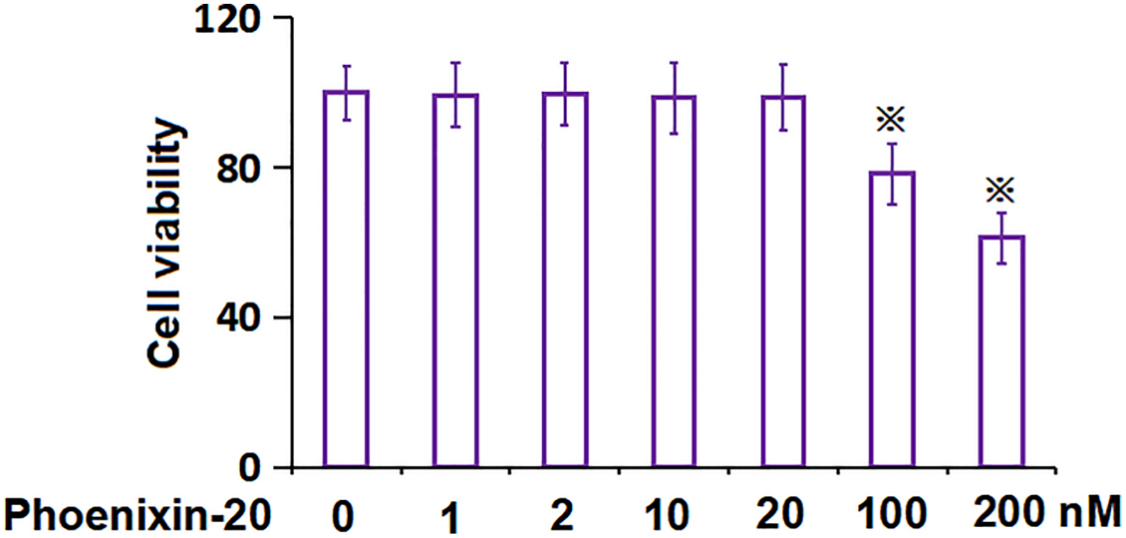
**The impact of Phoenixin-20 on the cell viability of RA-FLSs.** The cell viability of RA-FLSs was measured with CCK-8 following treatment with 0, 1, 2, 10, 20, 100, and 200 nM Phoenixin-20 (^※^*P* < 0.01 vs. vehicle group; ^#^, ^##^, *P* < 0.05, 0.01 vs. TNF-α group, *N* = 6).

To settle down the PNX-20 concentration to be incubated in RA-FLSs, cells were treated with 0, 1, 2, 10, 20, 100, and 200 nM PNX-20. As PNX-20 concentration was elevated from 0 to 20 nM, the cell viability was kept nearly unchanged, which was largely reduced to 78.3% and 61.2% by 100 and 200 nM PNX-20, respectively (Figure 2). Thus, 10 and 20 nM were picked as the concentrations utilized in the present study.

### PNX-20 repressed the inflammation in TNF-α-treated RA-FLSs

**Figure 3:**
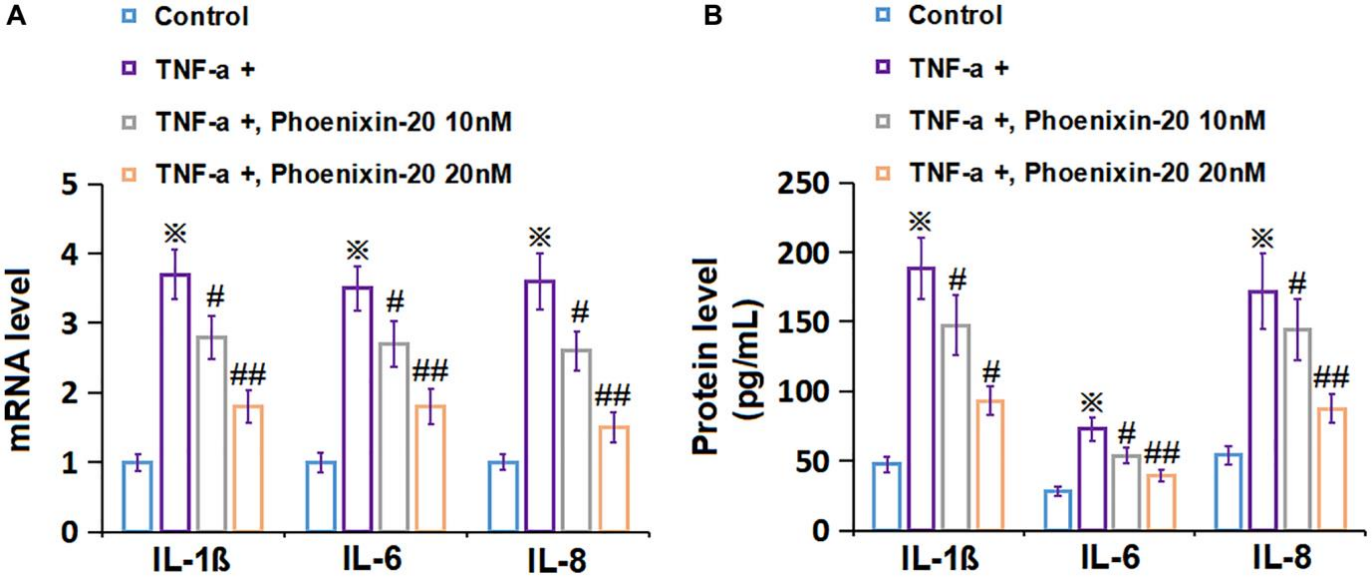
**Phoenixin-20 repressed the inflammation in TNF-α-treated RA-FLSs.** RA-FLSs were stimulated with TNF-α (10 ng/mL) with or without Phoenixin-20 (10, 20 nM) for 24 h. (**A**) mRNA level of IL-1β, IL-6, and IL-8. (**B**) Protein level of IL-1β, IL-6, and IL-8 (^※^*P* < 0.01 vs. vehicle group; ^#^, ^##^, *P* < 0.05, 0.01 vs. TNF-α group, *N* = 6).

RA-FLSs were handled with 10 ng/mL TNF-α, followed by introducing PNX-20 (10, 20 nM) or not for 1 day. The gene level of IL-1β, IL-6, and IL-8 was signally increased in TNF-α-treated RA-FLSs, which was markedly repressed by 10 and 20 nM PNX-20 (Figure 3A). Moreover, the IL-1β level in RA-FLSs was amplified from 47.5 to 188.1 pg/mL by TNF-α, which was markedly reduced to 147.5 and 92.9 pg/mL by 10 and 20 nM PNX-20, respectively. The IL-6 levels in the control, TNF-α, 10 nM PNX-20, and 20 nM PNX-20 groups were 28.2, 72.9, 53.7, and 39.4 pg/mL, respectively. Furthermore, the IL-8 level in RA-FLSs was elevated from 54.2 to 171.9 pg/mL by TNF-α, which was notably decreased to 144.6 and 87.7 pg/mL by 10 and 20 nM PNX-20, respectively (Figure 3B). A suppressive effect of PNX-20 on inflammation in TNF-α-treated RA-FLSs was observed.

### PNX-20 ameliorated TNF-α-induced cellular senescence in RA-FLSs

**Figure 4:**
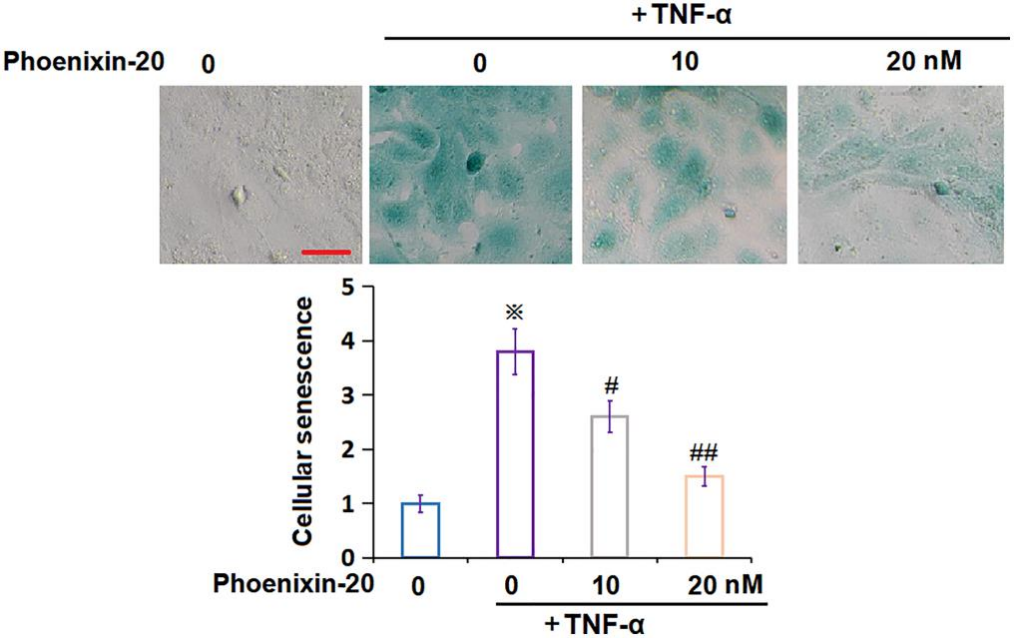
**Phoenixin-20 ameliorated TNF-α-induced cellular senescence in RA-FLSs.** RA-FLSs were stimulated with TNF-α (10 ng/mL) with or without Phoenixin-20 (10, 20 nM) for 7 days. Cellular senescence was assayed using SA-β-gal staining. Scale bar, 100 μm (^※^*P* < 0.01 vs. vehicle group; ^#^, ^##^, *P* < 0.05, 0.01 vs. TNF-α group, *N* = 6).

To check the impact of PNX-20 on the cellular senescence of TNF-α-treated RA-FLSs, RA-FLSs were handled with 10 ng/mL TNF-α, followed by introducing PNX-20 (10, 20 nM) or not for 7 days. The SA-β-gal staining assay was performed. The number of SA-β-gal positive RA-FLSs was observably increased by TNF-α, which was prominently reduced by 10 and 20 nM PNX-20 (Figure 4), implying a repressive property of PNX-20 against cellular senescence in TNF-α-treated RA-FLSs.

### PNX-20 attenuated TNF-α-induced reduction of telomerase activity in RA-FLSs

**Figure 5:**
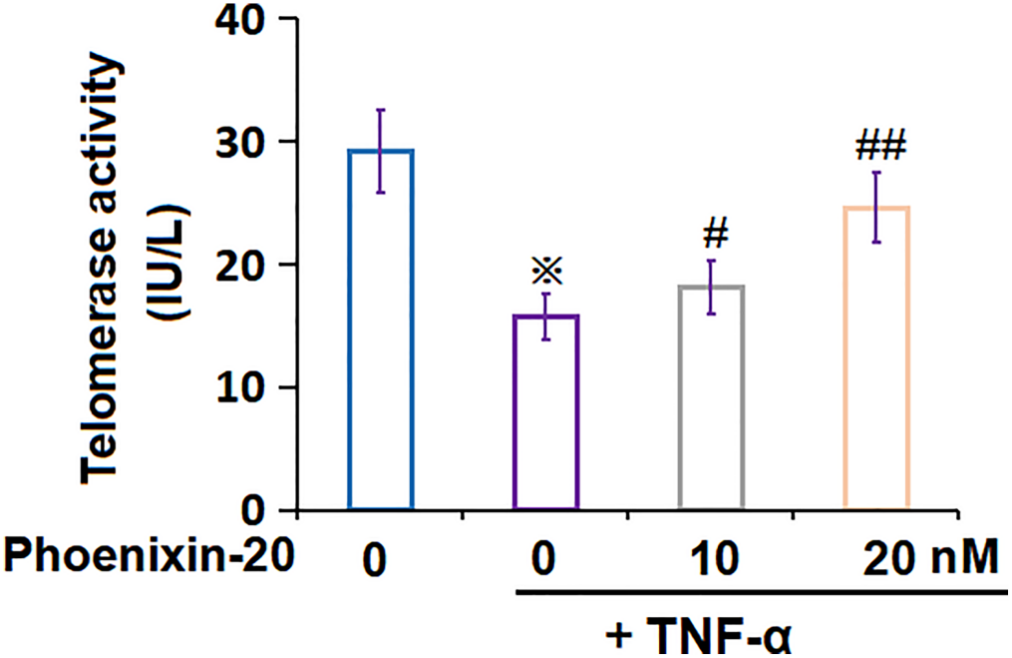
**Phoenixin-20 attenuated TNF-α-induced reduction of telomerase activity in RA-FLSs.** RA-FLSs were stimulated with TNF-α (10 ng/mL) with or without Phoenixin-20 (10, 20 nM) for 7 days. Telomerase activity was measured (^※^*P* < 0.01 vs. vehicle group; ^#^, ^##^, *P* < 0.05, 0.01 vs. TNF-α group, *N* = 5–6).

Telomerase activity is another biomarker for cellular senescence [22]. The telomerase activity in RA-FLSs declined from 29.2 to 15.7 pg/mL by TNF-α, which was markedly amplified to 18.1 and 24.6 IU/L by 10 and 20 nM PNX-20, respectively (Figure 5), implying an activation property of PNX-20 on telomerase activity in TNF-α-treated RA-FLSs.

### PNX-20 downregulated p53 and p21 in TNF-α-treated RA-FLSs

**Figure 6:**
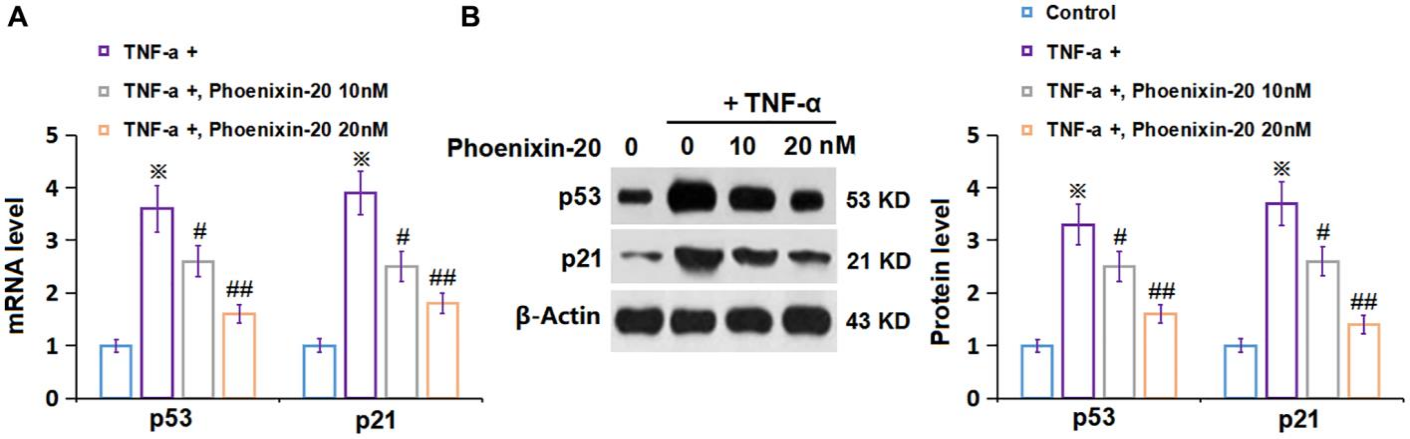
**Phoenixin-20 downregulated p53 and p21 in TNF-α-treated RA-FLSs.** RA-FLSs were stimulated with TNF-α (10 ng/mL) with or without Phoenixin-20 (10, 20 nM) for 7 days. (**A**) mRNA of p53 and p21; (**B**) Protein of p53 and p21 (^※^*P* < 0.01 vs. vehicle group; ^#^, ^##^, *P* < 0.05, 0.01 vs. TNF-α group, *N* = 5–6).

To explore the potential molecular mechanism of PNX-20 against cellular senescence, the level of biomarkers of cellular senescence was checked. P53 and p21 levels in RA-FLSs were notably elevated by TNF-α, which were markedly repressed by 10 and 20 nM PNX-20 (Figure 6A, 6B).

### PNX-20 repressed the expression of STAT6 in TNF-α-treated RA-FLSs

**Figure 7:**
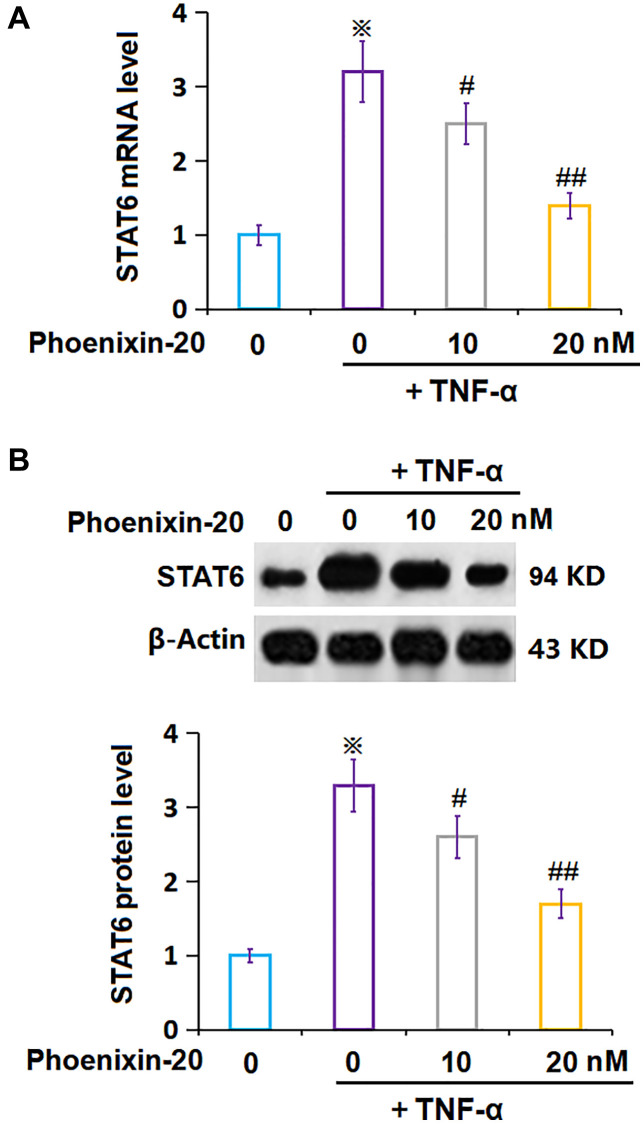
**Phoenixin-20 repressed the expression of STAT6 in TNF-α-treated RA-FLSs.** (**A**) mRNA of STAT6; (**B**) Protein of STAT6 (^※^*P* < 0.01 vs. vehicle group; ^#^, ^##^, *P* < 0.05, 0.01 vs. TNF-α group, *N* = 5–6).

STAT6 is a critical transcriptional factor regulating cellular senescence [23]. In TNF-α-treated RA-FLSs, STAT6 level was signally increased, which was markedly repressed by 10 and 20 nM PNX-20 (Figure 7A, 7B), suggesting that the function of PNX-20 might be correlated to STAT6.

### Overexpression of STAT6 abrogated the impact of PNX-20 on TNF-α-induced cellular senescence in RA-FLSs

**Figure 8:**
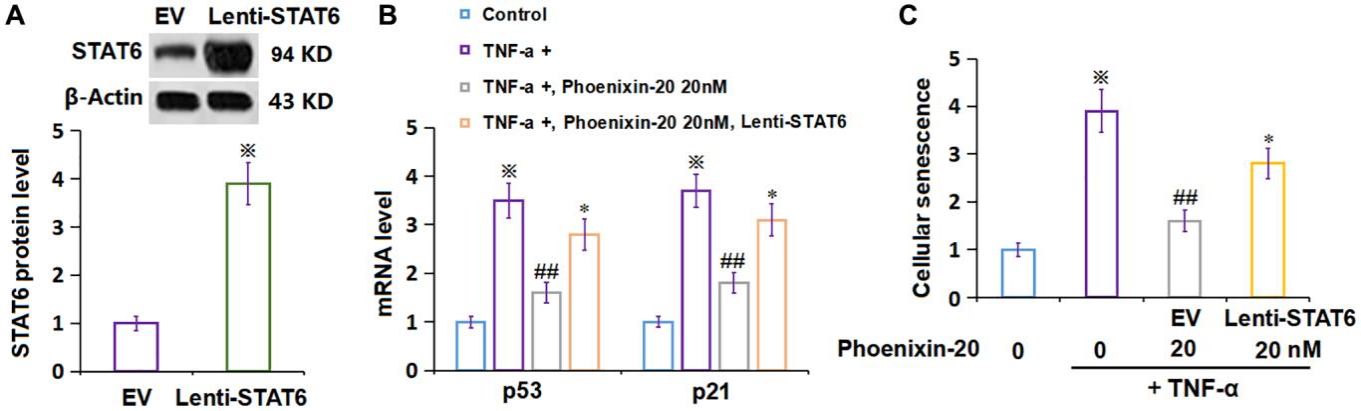
**Overexpression of STAT6 abolished the protective effect of Phoenixin-20 on TNF-α-induced cellular senescence in RA-FLSs.** Cells were transduced with lentiviral STAT6, followed by stimulation with TNF-α (10 ng/mL) and Phoenixin-20 (20 nM) for 7 days. (**A**) Western blot analysis revealed successful overexpression of STAT6; (**B**) mRNA of p53 and p21. (**C**) Cellular senescence was assayed using SA-β-gal staining (^※^*P* < 0.01 vs. vehicle group; ^##^*P* < 0.01 vs. TNF-α group, ^*^P < 0.05 vs. PNX-20 group, *N* = 5–6).

To confirm the molecular mechanism, RA-FLSs were transduced with lentiviral STAT6, followed by stimulation with TNF-α (10 ng/mL) and PNX-20 (20 nM) for 7 days. The overexpression of STAT6 in RA-FLSs was confirmed by Western blotting (Figure 8A). The increased level of p21 and p53 in TNF-α-treated RA-FLSs was markedly reduced by PNX-20, which was signally reversed by the overexpression of STAT6 in RA-FLSs (Figure 8B). Moreover, the amplified proportion of SA-β-gal positive cells observed in TNF-α-treated RA-FLSs was notably decreased by PNX-20, which was observably reversed by the overexpression of STAT6 in RA-FLSs (Figure 8C).

## DISCUSSION

A variety of immune cells are involved in the progression of RA, such as T, B cells, neutrophils, macrophages, and dendritic cells. Following activation by pathogenic factors, these immune cells secrete a variety of pro-inflammatory cytokines, including TNF-α, IL-1, IL-6, IL-8, etc. In addition to immune cells, inflammatory cytokines can be released by FLSs under RA conditions, which play a key role in disease persistence and progression of RA [24–26]. IL-1 inhibits proteoglycan synthesis and promotes bone resorption, while the secretion of collagenase and prostaglandin E2 (PGE2) is induced by the combined action of TNF-α and IL-1, which further accelerate bone resorption and bone destruction. Activated FLS release GM-CSF, which synergies with TNF-α and IL-8 to cause chemotaxis and activation of neutrophils [27]. Therefore, inhibition of differentiation and proliferation of FLSs may effectively slow the progression of RA and relieve clinical symptoms. Herein, the enhanced release of inflammatory cytokines in RA-FLSs was induced by TNF-α, which was in line with the research published by Wang [28]. After the introduction of PNX-20, the production of cytokines was markedly repressed, suggesting that the inflammation in RA-FLSs caused by TNF-α was notably inhibited by PNX-20, which indicated a protective function of PNX-20 on TNF-α-treated RA-FLSs.

Cell senescence was first recognized in the process of fibroblast proliferation induced by multi-generation culture, which was considered to be an effective way to prevent the infinite proliferation of tumor cells. Cell senescence has been confirmed to exist in various aging tissues and gradually accumulates over time, resulting in complex effects on the body [29]. With the gradual aging and degeneration, cellular senescence will be observed in chondrocytes and synovium cells in the joints, resulting in the decline of joint function. Cellular senescence affects the expression differences of various intracellular molecules, especially the upregulation of various inflammatory cytokines, epidermal growth factors, and matrix metalloproteinases, thus affecting cellular functions. These molecular phenotypes are known as SASP (senescence-associated secretory phenotype) [30]. In addition, the typical features of cellular senescence include elevated intracellular reactive oxygen species (ROS), upregulation of cell cycle inhibitors such as p16, p53, and p21, and increased SA-β-Gal activity [31]. Although cell senescence is considered to be one of the mechanisms to prevent the progression of precancerous lesions, the long-term existence of senescent cells in tissues seems to be harmful to healthy organisms [32]. A recent study has reported that cell senescence participates in the pathogenesis and progression of RA [33]. Herein, cellular senescence was observed in TNF-α-treated RA-FLSs, which was also reported by Zhang [34] in TNF-α-treated FLSs under the condition of hypoxia/reoxygenation. The cellular senescence of FLSs was markedly alleviated following the introduction of PNX-20, implying that the protective function of PNX-20 might be correlated to the repression of cellular senescence.

STAT6 is an important member of the STAT family, which mainly produces biological reactions in the downstream of IL-4 and IL-13. STAT6 consists of 847 amino acids, of which residue 641 is tyrosine and its phosphorylation plays a key role in IL-4-induced JAK-STAT signaling [35]. It is recently claimed that STAT6 participates in the progression of cellular senescence [23]. Herein, STAT6 was found downregulated in TNF-α-treated RA-FLSs, the level of which was largely reversed by PNX-20, implying that the function of PNX-20 on TNF-α-treated RA-FLSs might be correlated to the inhibition of STAT6. Moreover, the impact of PNX-20 on TNF-α-induced cellular senescence in RA-FLSs was abrogated by the overexpression of STAT6, further confirming the involvement of STAT6 in the function of PNX-20. In future work, the therapeutic function of PNX-20 against RA will be validated by an animal model.

Collectively, our study reveals that PNX-20 protected the TNF-α-induced cell senescence in RA-FLSs by downregulating STAT6. Based on these findings, we speculate that PNX-20 might be a promising agent for the treatment of RA.
